# Routinely staging gastric cancer with ^18^F-FDG PET-CT detects additional metastases and predicts early recurrence and death after surgery

**DOI:** 10.1007/s00330-018-5904-2

**Published:** 2019-01-14

**Authors:** John M. Findlay, Stefan Antonowicz, Ashvina Segaran, Jihene el Kafsi, Alexa Zhang, Kevin M. Bradley, Richard S. Gillies, Nicholas D. Maynard, Mark R. Middleton

**Affiliations:** 1 Oxford OesophagoGastric Centre, Churchill Hospital, Oxford University Hospitals NHS Foundation Trust, Oxford, OX3 7LE UK; 2 Department of Oncology, Old Road Campus Research Building, University of Oxford, Oxford, OX3 7DQ UK; 3 Department of Nuclear Medicine, Churchill Hospital, Oxford University Hospitals NHS Foundation Trust, Oxford, OX3 7LE UK

**Keywords:** Cancer staging, PET-CT, Gastric cancer, Prognosis, Metastases

## Abstract

**Objectives:**

Fluorodeoxyglucose (FDG) positron emission tomography–computed tomography (PET-CT) is typically considered to have minimal yield in gastric cancer, and so is not consistently recommended by international guidelines. However, its yield is considerable in esophageal and junctional cancer, identifying unsuspected metastases and risk-stratifying patients using metabolic nodal stage (mN). We aimed to determine the contemporary utility of routine ^18^F-FDG PET-CT in gastric cancer.

**Methods:**

We routinely stage patients with non-junctional gastric cancer with PET-CT, provided initial CT does not demonstrate unequivocal metastases. We performed a retrospective study of all such patients staged in our institution from January 2007 to July 2016. Our primary endpoint was detection of incurable disease. Our secondary endpoint was disease-free survival following gastrectomy. Decision theory, economic, and predictive models were generated.

**Results:**

The primary tumor was FDG-avid in 225/279 patients (80.6%). Seventy-two (25.8%) had FDG-avid nodes (resectable by D2 lymphadenectomy). This was not influenced by the Lauren classification. Unsuspected metastases were identified in 20 patients (7.2%). In 13 (4.7%), these would not have been otherwise identified. Decision theory and economic modeling supported routine PET-CT. Patients with FDG-avid nodes were more likely to have incurable disease (51.4% versus 15.5%; *p* < 0.001), and a worse prognosis if not: multivariate hazard ratio 2.19 (1.23–3.91; *p* = 0.008). Prognosis worsened with mN stage.

**Conclusions:**

PET-CT appears useful when used routinely for non-junctional gastric cancer, and should be considered in international recommendations. Any extra costs appear small and offset by avoiding futile investigations and radical treatment. mN stage identifies patients at risk of early recurrence and death.

**Key Points:**

*• PET-CT is typically not considered useful when staging gastric cancer. We describe a retrospective study of 279 patients routinely staged with PET-CT in the absence of metastases on CT.*

*• The primary tumor was avid in 80% of patients. Twenty-five percent had resectable avid nodes. PET-CT identified previously unsuspected metastases in 7% of patients, which would likely not have been identified by conventional staging without PET-CT in 5%. These patients were much more likely to have avid nodes.*

*• Beyond avoiding futile investigations and radical treatment in this 5%, we found patients with FDG-avid nodes (metabolic nodal stage, mN) to have a worse disease-free survival after gastrectomy.*

**Electronic supplementary material:**

The online version of this article (10.1007/s00330-018-5904-2) contains supplementary material, which is available to authorized users.

## Introduction

The contemporary utility of ^18^F-fluorodeoxyglucose (FDG) positron emission tomography–computed tomography (PET-CT) in gastric cancer is unclear. While in esophageal and gastro-esophageal junctional (GOJ) cancer it has clear utility in identifying metastases [1, 2], experience in gastric cancer is limited to two small studies of locally advanced disease from Korea and America. This lack of experience is perhaps due to initial reports that gastric cancer (particularly the diffuse subtype) is frequently not avid [3]. PET-CT is therefore not recommended routinely in Europe [3, 4].

We recently reported a novel aspect of PET-CT’s utility in esophageal and GOJ cancer: metabolic nodal stage (mN). We found that patients with FDG-avid nodes within a standard lymphadenectomy field had a higher risk of disease progression before surgery and recurrence and death afterwards [5–7]. However, whether this is true in gastric cancer is unclear, previously assessed by just two studies of Korean and Chinese populations [8, 9].

Radical treatment risks death and complications, and despite ostensibly curative surgery, recurrence remains common [10]. There is therefore an urgent need to improve staging to prevent futile treatment and develop prognostic markers. We have routinely staged patients with esophageal and gastric cancer with PET-CT for 10 years in the absence of unequivocal metastases on CT and conducted the largest retrospective study to date to determine its utility in a Western population.

## Materials and methods

### Study design and ethical approval

We included all patients with non-GOJ gastric adenocarcinoma without unequivocal metastases on CT from January 2007 to July 2016, following institutional Research and Development committee approval.

### Staging

All examinations were reported and reviewed at multidisciplinary team (MDT) meetings by specialist gastrointestinal radiologists, using the contemporary TNM 6th [11] or 7th [12] editions. PET-CT examinations were dual reported independently by two dedicated high-volume PET-CT radiologists, trained in nuclear medicine and clinical radiology*.* Patients were staged initially with a contrast-enhanced multidetector CT chest abdomen and pelvis as previously reported [13], followed by PET-CT and laparoscopy for disease more advanced than Tx/T1. Endoscopic ultrasound (EUS) was performed selectively.

PET-CT was performed before November 2009 using a GE Discovery STE 16-slice (60 min post 400 MBq ^18^F-FDG); after November 2009, a GE Discovery 690 64-slice system then also a GE Discovery 710 from September 2014, (both 90 min post 4 MBq/Kg FDG) all using ordered subset expectation maximization (OSEM) reconstruction, from skull base to upper thighs at 4 min per bed; after November 2014, a Bayesian penalized likelihood (BPL) reconstruction technique was used (Q.Clear) without intravenous contrast.

### Data

The following data were collected: patient gender and age; tumor location, grade of differentiation [12] and Lauren classification [14]; contemporaneous TNM stage; tumor maximum standardized uptake value (SUVmax) with that above background mediastinal blood pool being positive, the presence and number of FDG-avid nodes (mM1 1–2 nodes, mN2 > 2), defined as areas of avidity within a CT correlate suggesting a node visible separately from the primary tumor, within standard lymphadenectomy fields [13]; treatment strategy and neoadjuvant chemotherapy (NAC) regimen; restaging results after NAC; pathological stage [12], and response to NAC (Mandard Tumor Regression Grade 1–3 being response [15]); and recurrence and death (censored on 1st September 2017) .

Clinical T stage was grouped pragmatically as x/1, 2/3, or 4 according to the TNM 7th edition, and *N* as 0 or 1 to allow synthesis of data spanning the 6th and 7th editions (which had major differences in classification). Where available, EUS was used. Pathological stage was converted to TNM 7. Metastatic disease was defined as that outside a standard resection and D2 lymphadenectomy field, or invasion into unresectable structures. Metastatic disease evidenced on PET-CT was defined as such an area of avidity with a robust structural correlate. Suspicious isolated foci of avidity without a correlate were further investigated with cross-sectional imaging, direct visualization, or histopathology. Metastatic disease on imaging was subject to MDT consensus, unresectability at surgery by frozen section or consensus of two consultant esophagogastric surgeons.

### Neoadjuvant chemotherapy and restaging

Patients received 3 or 4 cycles of pre- +/− post-operative epirubicin, cisplatin, and 5-fluorouracil (ECF); epirubicin, cisplatin, and capecitabine (ECX); or epirubicin, oxaliplatin, and 5-fluorouracil ± bevacizumab as previously described, depending on contemporaneous practice and trials [5]. Patients were restaged with CT or PET-CT depending on local availability or trial protocols.

### Surgery

Total/subtotal gastrectomy was performed open or laparoscopically with D2 lymphadenectomy.

### Follow-up

Patients were reviewed clinically at 2 and 6 weeks after surgery, 3 monthly to one year, and 6–12 monthly thereafter for at least 5 years. Investigations for recurrence were performed only when suspected, typically involving cross-sectional imaging with CT or PET-CT ± endoscopy.

### Statistical analysis

Analyses were performed using R v3.0.2 [16]. Groups were compared using Fisher’s exact test and non-parametric data using the Mann-Whitney *U* test. *p* values were corrected using the Bonferroni technique for group and univariate analysis [17]. Multivariate binary logistic and Cox regression were performed for variables with *p* < 0.1, having excluded perfect separators. Logistic regression and recursive partitioning models were generated as previously described [2]. Survival metrics were calculated using the Kaplan-Meier method, follow-up using the reverse Kaplan-Meier method.

Probability thresholds were generated as previously described [2, 18], using metrics from this study or from the literature if unavailable: false positive rate 0.22% (risk of investigation of false positives assumed to be minimal for fine needle aspiration [19] and 0.47% for mediastinoscopy [20]), sensitivity 49.3%, 90-day mortality of surgery 1.66%, 2-year survival 52.4%, and lifetime attributable cancer risk from 13-mSv radiation dose 0.067% [21].

An economic model was developed using 2017 UK National Health Service (NHS) tariffs for laparoscopy (£735), 3 cycles ECX (£8004), gastrectomy (£10,402), PET-CT (£980), liver MRI (£145), mediastinoscopy (£1432), and ultrasound-guided fine needle aspiration (£599). A 37.6% likelihood of NAC and consequent restaging PET-CT was assumed.

## Results

Two hundred seventy-nine consecutive patients were identified (Table 1). EUS was performed in 61 (21.9%), laparoscopy in 220 (79.9%).

**Table 1:** Patient and tumor characteristics

Patients	Total 279
Age (median)	71.0 (IQR 62.3–78.0)
Gender	
Male (%)	185 (66.3)
Female (%)	94 (32.7)
Tumor	
Cell type	
Intestinal (%)	138 (49.5)
Diffuse (%)	86 (30.8)
Mixed (%)	26 (9.32)
Unknown (%)	29 (10.4)
Grade of differentiation	
Well (%)	13 (4.66)
Moderate (%)	61 (29.9)
Poor (%)	195 (69.9)
Unknown (%)	10 (2.58)
Site	
Proximal (%)	66 (23.7)
Body (%)	65 (23.3)
Distal (%)	134 (48.0)
Linitis (%)	14 (5.02)
Staging	
T stage	
x/1 (%)	57 (20.4)
2–3 (%)	165 (59.1)
4 (%)	54 (19.4)
Unknown (%)	3 (1.08)
N stage	
0 (%)	130 (46.6)
≥ 1 (%)	136 (48.7)
Unknown (%)	3 (1.08)

*IQR*, interquartile range

### Tumor avidity

The primary tumor was FDG-avid in 225 cases (80.6%). Seventy-two (25.8%) had FDG-avid nodes. An avid tumor was more likely with advancing T stage: T2–3 multivariate OR 3.38 (2.28–5.02; *p* = 0.002) and T4 7.46 (3.38–14.7; *p* = 0.003). There were no associations with the Lauren classification or differentiation (supplementary Table 1). Specifically, 61/86 (70.9%) of diffuse tumors were avid, although less so than intestinal: median SUVmax 5.10 (IQR 2.50–8.10) versus 8.90 (5.05–15.4; *p* < 0.001). There were no associations with nodal avidity or PET-CT system/reconstruction technique (supplementary Tables 1–2).

### Metastases on PET-CT

PET-CT identified possible or unequivocal metastatic disease in 40 patients (14.3%). In 14 (5.02%), this confirmed those suspected on prior CT. In 26 (9.32%), this was unsuspected. Of these 26, PET-CT was unequivocal in 12 (4.30%) and indeterminate in 14. These 14 were investigated further, with metastases confirmed in 8 (2.87%; *n* = 7 laparoscopy, *n* = 1 fine needle aspiration) and refuted in 6 (2.15%; *n* = 4 laparoscopy, *n* = 1 liver MRI, *n* = 1 mediastinoscopy). In a further 5 (1.79%), PET-CT refuted metastases suggested by CT.

In total, PET-CT identified unsuspected metastases in 20 patients (7.17%): *n* = 5 peritoneal, *n* = 2 peritoneal + liver/nodal, *n* = 5 liver, *n* = 1 liver + nodal, *n* = 6 nodal, and *n* = 1 nodal + skeletal. In 13 of these 20 (4.66%), these metastases would not have been visible at subsequent laparoscopy (for example retroperitoneal/mediastinal/cervical nodes, skeletal, or deep liver metastases). In patients without metastatic disease on PET-CT, this was subsequently identified in 35 (false negatives; 12.5%; *n* = 34 at laparoscopy, *n* = 1 at surgery without NAC).

Following complete staging, there were 34 true positives (evident on PET-CT, either unequivocal or subsequently confirmed), 6 false positives (possible metastases subsequently refuted), 35 false negatives (identified following PET-CT), and 204 true negatives (no metastatic disease following staging or surgery without NAC). PET-CT was 49.3% sensitive (37.0–61.6) and 97.1% specific (93.9–98.9) for metastatic disease. Positive predictive value (PPV) was 85.0% (71.3–92.8%); negative predictive value (NPV) was 85.4% (82.2–88.1%).

### Factors associated with metastases on PET-CT

Of factors available before PET-CT, both advancing T and N stage were associated with true positive metastases on PET-CT (supplementary Table 3). Only N ≥ 1 stage remained significant on multivariate analysis (Table 2). When PET-CT factors were considered, only the presence of FDG-avid nodes was predictive: multivariate OR 3.97 (1.33–11.9; *p* = 0.014). This was independent of clinical N ≥ 1 stage, which was borderline significant (Table 2).

**Table 2:** Multivariate regression: factors associated with metastases

Characteristic	Odds ratio (95% CI)	*p*
Metastases on PET-CT: pre-PET-CT variables		
Age	0.99 (0.95–1.03)	0.599
Gender		
Female	Reference	Reference
Male	0.59 (0.22–1.59)	0.297
Tumor site		
Proximal	Reference	Reference
Body	0.77 (0.23–2.60)	0.669
Distal	0.64 (0.21–1.96)	0.438
Linitis	0.80 (0.07–8.58)	0.854
Lauren classification		
Intestinal	Reference	Reference
Diffuse	1.94 (0.67–5.67)	0.224
T stage		
x/1	Reference	Reference
2/3	0.57 (0.13–2.51)	0.454
4	1.97 (0.42–9.31)	0.393
N stage		
0	Reference	Reference
≥ 1	5.86 (1.67–20.6)	0.006
Metastases on PET-CT: all variables		
Age	1.00 (0.95–1.04)	0.926
Gender		
Female	Reference	Reference
Male	0.55 (0.19–1.55)	0.260
Tumor site		
Proximal	Reference	Reference
Body	1.05 (0.28–3.88)	0.945
Distal	0.62 (0.19–2.06)	0.438
Linitis	1.22 (0.09–15.9)	0.881
Lauren classification		
Intestinal	Reference	Reference
Diffuse	1.19 (0.37–2.81)	0.764
T stage		
x/1	Reference	Reference
2/3	0.43 (0.09–2.06)	0.293
4	1.56 (0.30–8.14)	0.599
N stage		
0	Reference	Reference
≥ 1	3.78 (0.96–15.0)	0.058
mN		
0	Reference	Reference
≥ 1	3.97 (1.33–11.9)	0.014
SUVmax	1.04 (0.99–1.10)	0.148

Twenty-two of 72 (30.6%) patients with FDG-avid nodes had metastatic disease on PET-CT, compared with 12/207 (5.80%) without (*p* < 0.001). This association persisted for ≥ 2 avid nodes (mN2 stage; multivariate OR 7.11 [3.84–13.2] *p* = 0.001) rather than ≤ 2 (mN1 stage; OR 0.96 [0.38–2.41] *p* = 0.956); 18 of 45 (40.0%) versus 4 of 27 (14.8%), respectively (*p* < 0.001).

### Factors associated with metastases at staging overall

Overall, metastases were more likely with worsening differentiation, T and N stage, tumor site (linitis and proximal tumors), and the presence of FDG-avid nodes (supplementary Table 3). On multivariate analysis, only FDG-avid nodes remained significant (Table 3).

**Table 3:** Multivariate regression: factors associated with metastases at staging

Characteristic	Odds ratio (95% CI)	*p*
Metastases at staging (PET-CT or laparoscopy)		
Age	0.99 (0.96–1.03)	0.582
Gender		
Female	Reference	Reference
Male	0.93 (0.39–2.18)	0.861
Tumor site		
Proximal	Reference	Reference
Body	0.89 (0.31–2.95)	0.823
Distal	0.43 (0.16–1.17)	0.097
Linitis	4.46 (0.92–21.7)	0.064
Lauren classification		
Intestinal	Reference	Reference
Diffuse	0.86 (0.35–2.08)	0.731
T stage		
x/1	Reference	Reference
2/3	0.95 (0.31–2.88)	0.921
4	1.05 (0.27–4.10)	0.949
N stage		
0	Reference	Reference
≥ 1	1.39 (0.52–3.69)	0.508
mN		
0	Reference	Reference
≥ 1	3.92 (1.40–11.0)	0.009
SUVmax	0.94 (0.88–1.02)	0.125
Unsuspected metastases at laparoscopy		
Age	1.01 (0.97–1.07)	0.545
Gender		
Female	Reference	Reference
male	0.98 (0.36–2.62)	0.961
Tumor site		
Proximal	Reference	Reference
Body	0.67 (0.19–2.41)	0.543
Distal	0.39 (0.12–1.20)	0.101
Linitis	3.78 (0.64–22.4)	0.143
Lauren classification		
Intestinal	Reference	Reference
Diffuse	0.58 (0.21–1.61)	0.300
T stage		
x/1	Reference	Reference
2/3	2.25 (0.54–9.33)	0.262
4	11.82 (0.31–10.6)	0.505
N stage		
0	Reference	Reference
≥ 1	2.57 (0.95–5.42)	0.064
mN		
0	Reference	Reference
≥ 1	5.19 (1.50–18.0)	0.009
SUVmax	0.96 (0.88–1.04)	0.280

Thirty-seven of 72 (51.4%) patients with FDG-avid nodes had metastases, compared with 32/207 (15.5%) without (*p* < 0.001). Again, risk was most for patients with mN stage 2: 28/45 (62.2%) versus 9/27 with mN1 (33.3%; *p* < 0.0001). Indeed, on multivariate analysis, just mN2 stage remained significant: OR 6.15 (2.26–16.8; *p* < 0.001).

### Factors associated with unsuspected metastases at laparoscopy/surgery without chemotherapy

Almost identical associations were seen for unsuspected metastases at surgery (i.e., false negatives on PET-CT; supplementary Table 4). On multivariate analysis, only FDG-avid nodes persisted (Table 3).

Fifteen of 45 (33.3%) patients with FDG-avid nodes on PET-CT (but no apparent metastases) had unsuspected metastases compared with 20/195 without (10.3%; *p* = 0.001). Risk increased with mN stage: mN1 OR 4.49 (1.03–19.5; *p* = 0.045) and mN2 OR 6.03 (1.38–26.3; *p* = 0.017).

### Decision theory

The probability threshold for PET-CT was 1.90%, i.e., the pre-PET-CT probability of finding metastases at which its risk (radiation and the implications of false positives and false negatives) justifies its potential benefit [2]. No models could identify patients below this to forgo PET-CT.

### Economic model

An economic model was developed to calculate net staging costs, assuming PET-CT identified unsuspected metastases in 7 of 279 patients which might have been identified at laparoscopy, plus 13 in whom these would not. This would save 9 staging laparoscopies (as in 11 cases, laparoscopy was performed to confirm/refute findings), plus 13 futile attempts at treatment (NAC, restaging plus gastrectomy). Possible metastases resulted in one additional liver MRI, mediastinoscopy, and percutaneous FNA. The net additional cost was £322.01 per patient, and £6910.86 to avoid one futile attempt at radical treatment.

### Prognosis before treatment

One hundred sixty patients underwent successful surgery (85 after NAC, 75 without). Median OS was 1585 days (IQR 1250, not reached). Median DFS was 1408 days (967–1849). Median follow-up was 1478 days (1360–1739). On multivariate Cox regression analysis of pre-treatment factors, only the presence of FDG-avid nodes at staging was associated with DFS: HR 2.36 (1.33–4.19; *p* = 0.003). Prognosis worsened with mN stage (Table 4; supplementary Table 6).

**Table 4:** Multivariate Cox regression analysis before surgery: disease-free survival

Characteristic	HR (95% CI)	*p*
Pre-treatment variables, all patients		
T stage		
x/1	Reference	Reference
2/3	1.88 (0.98–3.60)	0.059
4	1.60 (0.66–3.87)	0.296
mN		
0	Reference	Reference
1	2.06 (1.05–4.03)	0.035
2	3.48 (1.33–9.12)	0.011
Pre-surgery variables, all patients		
T stage		
x/1	Reference	Reference
2/3	1.67 (0.87–3.22)	0.126
4	1.30 (0.53–3.21)	0.567
mN		
0	Reference	Reference
1	1.89 (0.96–3.70)	0.065
2	3.44 (1.29–9.22)	0.014
NAC		
No	Reference	Reference
Yes	2.06 (1.25–3.38)	0.004
Pre-surgery variables, patients with definitive pre-operative mN stage		
T stage		
x/1	Reference	Reference
2/3	1.29 (0.54–3.09)	0.564
4	1.20 (0.37–3.95)	0.762
mN		
0	Reference	Reference
≥ 1	2.77 (1.12–6.83)	0.027
NAC		
No	Reference	Reference
Yes	3.99 (2.08–7.66)	< 0.001
Pre-surgery variables, patients with definitive pre-operative mN stage		
T stage		
x/1	Reference	Reference
2/3	1.45 (0.58–3.62)	0.428
4	1.03 (0.30–3.53)	0.996
pN mN		
pN0 mN0	Reference	Reference
pN1 mN0	2.44 (1.20–4.96)	0.013
pN1 mN1	3.87 (1.35–11.1)	0.012
NAC		
No	Reference	Reference
Yes	3.55 (1.83–6.89)	< 0.001

### Prognosis before surgery

**Fig. 1:**
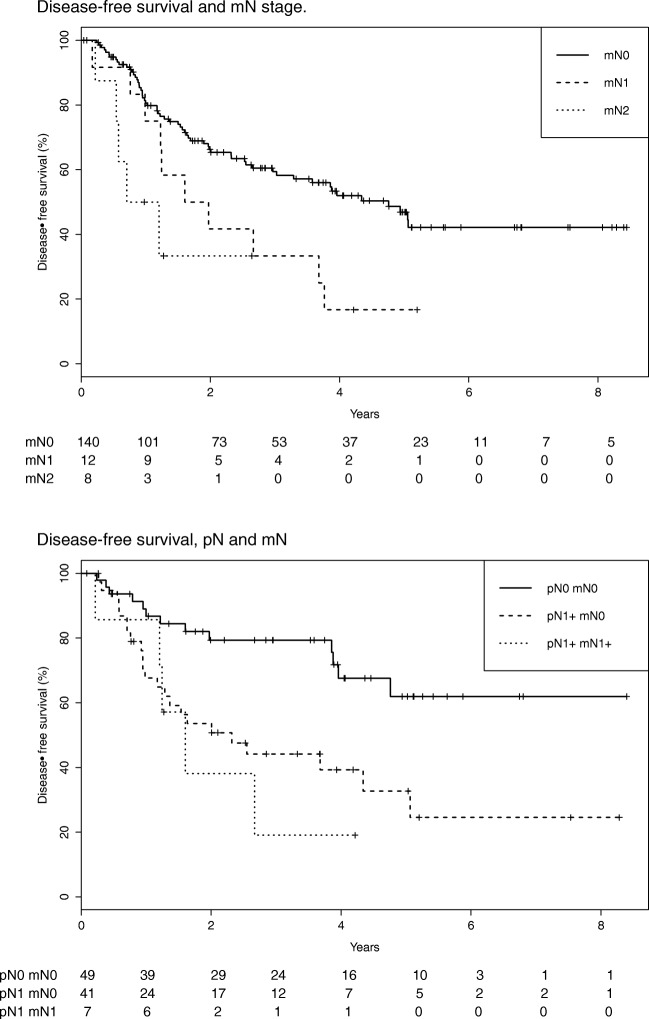
Disease-free survival and mN stage

Adjusting for NAC, the presence of FDG-avid nodes at staging remained independent: HR 2.19 (1.23–3.91; *p* = 0.008). Again, prognosis worsened with advancing mN stage (Table 4; Fig. 1). This association was refined when considering those patients with definitive pre-operative mN stage (*n* = 75 patients not receiving NAC, *n* = 24 patients receiving NAC and restaged by PET-CT): multivariate HR 2.77 (1.12–6.83; *p* = 0.027).

### Prognosis after surgery

After surgery, a number of pathological factors were associated with DFS on univariate analysis (supplementary Table 7). On multivariable analysis, progressive pN and pathological response (but not mN) remained independent in patients receiving NAC (supplementary Table 8). In patients not receiving NAC, no factors were significant.

### Predicting DFS before treatment

A recursive partitioning model found FDG-avid nodes to be the most reliable method of identifying high-risk patients: just 6 of 12 patients (50.0%) were alive and disease-free at 2 years (0.00% at 5 years), compared with 71/115 (61.7%) without (23/71; 32.4%; *p* = 0.002).

Overall, the presence of FDG-avid nodes at staging was highly specific for death/recurrence at 2 years: 92.2% (83.8–97.1), with a PPV of 66.7% (44.4–83.4). Overall, sensitivity was low: 21.4% (11.6–34.4). At 5 years, specificity rose to 100.0% (85.2–100), with PPV rising to 72.3% (61.4–81.6). This was identical to the corresponding logistic regression model.

### Association between mN, EUS, and pN

Sixty-one patients were staged with EUS, suggesting nodal disease in 23 (37.7%). Nine had FDG-avid nodes (all with EUS-positive nodes). With respect to EUS nodal stage, there were 9 true positives, no false positives, 14 false negatives, and 38 true negatives, making the presence of FDG-avid nodes 39.1% sensitive (19.7–61.4%) and 100% specific (90.8–100%) for clinical nodal disease at EUS.

Of the 99 patients with a definitive pre-operative mN stage, 8 had FDG-avid nodes while 48 had pathologically involved nodes. All but one of these 8 had pathologically involved nodes (7 true positives, 1 false positive, 50 true negatives, and 41 false negatives), making the presence of FDG-avid nodes 14.6% (6.07–27.8) sensitive and 98.0% (89.6–100.0) specific for pathologically involved nodes. The single false negative patient developed early recurrence after 449 days, raising the possibility of a missed nodal metastasis.

For 98 patients, a hybrid pNmN stage could be generated. On multivariable analysis (Table 4), relative to pN0mN0 disease, patients with pN ≥ 1mN0 disease (i.e., non-avid nodal metastases) had a worse DFS (HR 2.44 [1.20–4.96]; *p* = 0.013). However, prognosis in those with avid nodal metastases (pN ≥ 1mN ≥ 1) was even worse (HR 3.87 [1.35–11.1]; *p* = 0.012; Fig. 1).

## Discussion

We found that routinely staging all patients with gastric cancer with ^18^F-FDG PET-CT was useful, both identifying unsuspected metastases and risk-stratifying patients. In 4.7% of patients, PET-CT identified unsuspected metastases which would not have been detected by conventional staging. One quarter of patients had FDG-avid nodes, which conferred a worse prognosis: a significantly higher risk of coincident incurable disease at staging and worse disease-free survival following surgery if curable (independent of clinical and pathological nodal stage). Utility was not influenced by the Lauren classification and was supported by decision theory and cost-effective in a limited economic model.

These findings build on a small number of retrospective studies. In a 2005 Korean study of 68 patients, Chen et al reported PET to improve staging by conferring greater sensitivity for identifying resectable metastases [22]; however, this study excluded patients with diabetes mellitus and performed PET without hybrid CT images and did not demonstrate greater sensitivity for distant metastases. Subsequently, Smyth et al reported selective PET-CT to identify occult metastases in 10% of non-consecutive patients with advanced (T3–4) gastric cancer in the USA, with a similarly favorable cost profile, although did not assess its utility in patients with less advanced disease and did not assess nodal avidity [23]. Some smaller studies have reported the presence of FDG-avid nodes to confer a worse prognosis, although in less defined cohorts. In 151 patients undergoing gastrectomy in Korea, Song et al found those with resectable FDG-avid nodes (SUVmax > 2.8) to have worse OS and DFS [8], although did not quantify mN stage. Moreover, this study assessed only patients with nodal involvement pathologically following surgery, by definition not assessing the utility of PET-CT in detecting metastases, and also excluding patients receiving NAC (the majority in clinical practice). In 2016, Wang et al assessed the latter in 50 patients in China, finding those with mN stage 2 had worse OS, but notably included patients undergoing non-curative surgery [9]. Coupe et al previously reported FDG-avid nodes to confer a worse prognosis in 68 patients with GOJ or gastric cancer, although did not assess the staging utility of PET-CT and also included patients undergoing non-curative treatment [24]. However, we believe ours to be the largest study to date, the first in a primarily Caucasian population of non-junctional cancer and also the first to demonstrate associations between avid nodes and metastatic disease. The latter suggests a mechanism by which FDG-avid nodes confer a worse prognosis, as a surrogate for a more aggressive and metastatic cancer phenotype. This is illustrated by the relative insensitivity but absolute specificity of PET-CT as regards nodal disease, suggesting the merit of PET-CT to be the identification of a subset of patients with nodal metastases with worse disease.

If validated, these novel findings have a number of implications. Firstly, they suggest that a sizable minority of patients when not staged by PET-CT have detectable metastatic disease and undergo futile radical therapy. Routine use of PET-CT might allow these patients to commence palliative therapies sooner and obviate the risks, complications, trauma, and costs of well-intentioned but ultimately futile treatment. Secondly, routine PET-CT may identify metastatic disease rendering laparoscopy and EUS unnecessary, similarly obviating their risks and costs (although both retain invaluable roles in patients without metastases evident on PET-CT). Thirdly, patients with FDG-avid nodes at staging may have a more aggressive phenotype.

This is independent of the mere presence of nodal metastases, suggesting FDG avidity is a useful surrogate biomarker. Such high-risk patients might be targeted for more intensive staging and restaging investigations. Finally, these patients (particularly those with a definitive mN stage before surgery) can be counseled more fully as to their prognosis, allowing them to make more informed decisions and undergo tailored surveillance.

These findings also highlight priorities for research. Our cohort was insufficient to determine whether restaging patients with PET-CT rather than CT is preferable, but intuitively the greater sensitivity of PET-CT should translate to restaging (as we previously reported in esophageal cancer) [13]. It is also unclear whether PET-CT may have similar utility in monitoring response of nodal metastases to NAC [6]. While we did not find mN stage to be an independent marker of prognosis once adjusting for pathological stage, this might be due to the limited number of patients with a definitive pre-operative mN stage.

We acknowledge our study to have a number of limitations and sources of bias; these include its retrospective nature—although by performing PET-CT routinely, we believe the potential for selection bias is minimal, and the results generalizable, although we acknowledge potential bias in the use of PET-CT to corroborate suspicious but equivocal metastases on prior CT; a study period spanning evolution in technology and clinical practice potentially introducing performance bias, as well as the need to consider cT2 and cT3 diseases as a single variable and its single-center nature. Consequently, prospective and external validation will be required. Our economic modeling was by necessity limited and conservative. We chose the UK NHS tariffs as a consistent and pragmatic estimate of costs; in reality, once additive costs are included, and most importantly those financial, physical and psychological costs incurred by patients and those supporting them, it is likely that the net savings of will be far greater. Beyond this, as our primary aim was to assess staging utility, we have included a number of patients with limited follow-up, with resultant detection bias and performance bias for prognostication. Also, by performing on-demand investigations for recurrence tailored to symptoms, it is again likely that some instances of recurrence in these patients are yet to be detected.

In summary, we found routine staging PET-CT to identify metastases in 7% of patient with non-junctional cancer, most of which would not be identified by conventional staging. This prevented patients receiving futile radical therapy, and its routine use was supported both by decision theory and limited economic modeling. Furthermore, PET-CT identifies patients with avid nodes who are at substantially higher risk of metastatic disease and a worse prognosis following radical treatment. mN stage therefore appears to be a novel and useful biomarker when staging non-junctional gastric cancer.

## Electronic supplementary material

ESM 1 (DOCX 38 kb)

## Abbreviations

CT: Computed tomography
DFS: Disease-free survival
ECX: Epirubicin, cisplatin, and capecitabine
EUS: Endoscopic ultrasound
FDG: Fluorodeoxyglucose
GE: General Electric
GOJ: Gastro-esophageal junction
IQR: Interquartile range
MDT: Multidisciplinary team
mN: Metabolic nodal stage
mNR: Metabolic nodal response
MRI: Magnetic resonance imaging
NAC: Neoadjuvant chemotherapy
NHS: National Health Service
NPV: Negative predictive value
OR: Odds ratio
OS: Overall survival
OSEM: Ordered subset expectation maximization
PET: Positron emission tomography
PPV: Positive predictive value
SUVmax: Maximum standardized uptake variable
