# Supplementary material for: Routinely staging gastric cancer with 18F-FDG PET-CT detects additional metastases and predicts early recurrence and death after surgery
Source: Eur Radiol. 2019 Jan 14;29(5):2490–8. doi: 10.1007/s00330-018-5904-2 (PMC6443603; doi:10.1007/s00330-018-5904-2)
Supplement: Supplementary file 1 — (DOCX 38 kb) [file 330_2018_5904_MOESM1_ESM.docx]

Supplementary table 1 – Characteristics of FDG-avid and non-avid tumours

| Characteristic | FDG-avid (n=225) | Not FDG-avid (n=54) | p  corrected α=0.006 |
| --- | --- | --- | --- |
| Age (median; IQR) | 71 (65-78) | 69.5 (60.3-77.8) | 0.230^a^ |
| Gender  *male*  *female* | 155 (68.9%)  70 (31.1%) | 30 (55.6%)  24 (43.6%) | 0.078^b^ |
| Cell subtype  *intestinal*  *diffuse*  *mixed*  *unknown* | 116 (51.6%)  61 (27.1%)  21 (9.33%)  27 (12.0%) | 22 (40.7%)  25 (46.3%)  5 (9.26%)  2 (3.70%) | 0.070^b^ |
| Grade of differentiation  *well*  *moderate*  *poor*  *unknown* | 12 (5.33%)  52 (23.1%)  152 (67.6%)  9 (4.00%) | 1 (1.85%)  9 (16.7%)  43 (79.6%)  1 (1.85%) | <0.001^b^ |
| Site  *proximal*  *body*  *distal*  *linitis* | 55 (24.4%)  53 (23.6%)  108 (48.0%)  9 (4.00%) | 11 (20.4%)  12 (22.2%)  26 (48.1%)  5 (9.26%) | 0.436^b^ |
| T stage  *x/1*  *2/3*  *4*  *unknown* | 35 (15.6%)  49 (21.8%)  138 (61.3%)  3 (1.33%) | 22 (40.7%)  5 (9.26%)  27 (50.0%)  0 (0.0%) | p<0.001^b^ |
| N stage  *0*  *≥1*  *unknown* | 105 (46.7%)  117 (52.0%)  3 (1.33%) | 41 (75.9%)  13 (24.1%)  0 (0.0%) | p<0.001^b^ |
| PET-CT system  *STE*  *690*  *710* | 48 (21.3%)  150 (66.7%)  27 (12.0%) | 16 (29.6%)  29 (53.4%)  9 (16.7%) | p=0.190^b^ |

a=Mann-Witney test b=Fisher’s exact test; IQR=interquartile range

Supplementary table 2 – PET-CT characteristics of tumour subtypes

| Characteristic | Primary tumour SUVmax  (median, IQR) | p | Nodal tumour avidity | p |
| --- | --- | --- | --- | --- |
| Cell subtype  *intestinal*  *diffuse*  *mixed* | 8.90 (5.05-15.4)  5.10 (2.50-8.10)  8.45 (4.53-12.4) | <0.001^a^ | 39/138 (28.3%)  15/86 (17.4%)  5/26 (19.2%) | 0.156^b^ |
| Differentiation  *well*  *moderate*  *poor* | 9.10 (5.40-16.5)  8.10 (5.00-14.6)  7.40 (4.05-12.7) | 0.222^a^ | 1/13 (7.69%)  13/51 (25.5%  57/195 (29.2%) | 0.150^b^ |
| PET-CT system  *STE*  *690*  *710* | 8.00 (205-16.6)  7.10 (4.40-13.7)  8.30 (4.38-16.8) | 0.546^a^ | 11/64 (17.2%)  52/179 (29.1%)  9/36 (34.6%) | 0.175^b^ |

a=Kruskal-Wallis test b=Fisher’s exact test; IQR=interquartile range

Supplementary table 3 – Factors associated with metastatic disease on PET-CT

| Characteristic | Metastatic disease (n=34) | No metastatic disease (n=245) | p  Corrected α=0.006 |
| --- | --- | --- | --- |
| Age (median; IQR) | 71.0 (63.5-75.0) | 71.0 (65.0-79.0) | 0.293^a^ |
| Gender  *male*  *female* | 22 (64.7%)  12 (35.3%) | 163 (66.5%)  82 (33.5%) | 0.848^b^ |
| Cell subtype  *intestinal*  *diffuse*  *mixed*  *unknown* | 18 (52.9%)  9 (26.5%)  0 (0.0%)  5 (14.7%) | 120 (49.0%)  77 (31.4%)  26 (10.6%)  22 (8.98%) | 0.145^b^ |
| Grade of differentiation  *well*  *moderate*  *poor*  *unknown* | 0 (0.0%)  7 (20.6%)  23 (67.6%)  4 (11.8%) | 13 (5.31%)  54 (22.0%)  172 (70.2%)  6 (2.45%) | 0.555^b^ |
| Site  *proximal*  *body*  *distal*  *linitis* | 13 (38.2%)  8 (23.5%)  10 (29.4%)  3 (8.82%) | 53 (21.6%)  57 (23.3%)  124 (50.6%)  11 (4.49%) | 0.045^b^ |
| T stage  *x/1*  *2/3*  *4*  *unknown* | 3 (8.82%)  14 (41.2%)  17 (50.0%)  0 (0.0%) | 54 (22.0%)  151 (61.6%)  37 (15.1%)  3 (1.22%) | p<0.001^b^ |
| N stage  *0*  *≥1*  *unknown* | 4 (11.8%)  30 (88.2%)  0 (0.0%) | 100 (40.8%)  142 (58.0%)  3 (1.22%) | p<0.001^b^ |
| SUVmax (median; IQR) | 11.9 (8.03-18.6) | 7.10 (4.00-12.4) | p<0.001^b^ |
| mN  *0*  *1*  *2* | 12 (35.3%)  4 (11.7%)  18 (52.9%) | 195 (79.6%)  23 (9.39%)  27 (11.0%) | p<0.001^b^ |

IQR=interquartile range; a=Wilcoxon signed ranks test; b=Fisher’s exact test

Supplementary table 4 – Factors associated with incurable disease at staging

| Characteristic | Metastatic disease (n=69) | No metastatic disease (n=210) | p  Corrected α=0.006 |
| --- | --- | --- | --- |
| Age (median; IQR) | 70 (64.3-76.0) | 71.0 (65.0-79.0) | 0.173^a^ |
| Gender  *male*  *female* | 43 (62.3%)  26 (37.7%) | 142 (67.6%)  68 (32.4%) | 0.464^b^ |
| Cell subtype  *intestinal*  *diffuse*  *mixed*  *unknown* | 31 (44.9%)  23 (33.3%)  2 (2.9%)  13 (18.8%) | 107 (51.0%)  63 (30.0%)  24 (11.4%)  16 (7.62%) | 0.119^b^ |
| Grade of differentiation  *well*  *moderate*  *poor*  *unknown* | 0 (0.0%)  9 (13.0%)  56 (81.2%)  4 (5.8%) | 13 (6.19%)  52 (24.8%)  139 (66.2%)  6 (2.86%) | 0.007^b^ |
| Site  *proximal*  *body*  *distal*  *linitis* | 22 (31.9%)  15 (21.7%)  22 (31.9%)  10 (14.5%) | 44 (21.0%)  50 (23.8%)  112 (53.3%)  4 (1.90%) | <0.001^b^ |
| T stage  *x/1*  *2/3*  *4*  *unknown* | 8 (11.6%)  37 (53.6%)  24 (34.8%)  0 (0.0%) | 49 (23.3%)  128 (61.0%)  30 (14.3%)  3 (1.43%) | <0.001^b^ |
| N stage  *0*  *≥1*  *unknown* | 17 (24.6%)  52 (75.4%)  0 (0.0%) | 78 (37.1%)  129 (61.4%)  3 (1.22%) | <0.001^b^ |
| SUVmax (median; IQR) | 8.70 (5.60-16.1) | 7.10 (4.00-12.7) | 0.020^a^ |
| mN  *0*  *1*  *2* | 32 (46.4%)  9 (13.0%)  28 (40.6%) | 175 (83.3%)  18 (8.57%)  17 (8.10%) | <0.001^b^ |

Supplementary table 5 – Factors associated with unsuspected incurable disease at laparoscopy or surgery without neoadjuvant therapy

| Characteristic | Metastatic disease (n=35) | No metastatic disease (n=210) | p  Corrected α=0.006 |
| --- | --- | --- | --- |
| Age (median; IQR) | 69.0 (64.5-77.5) | 71.0 (65.0-79.0) | 0.341^a^ |
| Gender  *male*  *female* | 21 (60.0%)  14 (40.0%) | 142 (67.6%)  68 (32.4%) | 0.440^b^ |
| Cell subtype  *intestinal*  *diffuse*  *mixed*  *unknown* | 13 (37.1%)  14 (40.0%)  2 (5.71%)  6 (17.1%) | 107 (51.0%)  63 (30.0%)  24 (11.4%)  16 (7.62%) | 0.269^b^ |
| Grade of differentiation  *well*  *moderate*  *poor*  *unknown* | 0 (0.0%)  2 (5.71%)  33 (94.3%)  0 (0.0%) | 13 (6.19%)  52 (24.8%)  139 (66.2%)  6 (2.86%) | 0.004^b^ |
| Site  *proximal*  *body*  *distal*  *linitis* | 9 (25.7%)  7 (20.0%)  12 (34.3%)  7 (20.0%) | 44 (21.0%)  50 (23.8%)  112 (53.3%)  4 (1.90%) | <0.001^b^ |
| T stage  *x/1*  *2/3*  *4*  *unknown* | 5 (14.3%)  23 (65.7%)  7 (20.0%)  0 (0.0%) | 49 (23.3%)  128 (61.0%)  30 (14.3%)  3 (1.43%) | p=0.391^b^ |
| N stage  *0*  *≥1*  *unknown* | 22 (62.9%)  13 (37.1%)  0 (0.0%) | 78 (37.1%)  129 (61.4%)  3 (1.22%) | p=0.009^b^ |
| SUVmax (median; IQR) | 6.60 (4.40-10.3) | 7.10 (4.00-12.7) | p=0.723^a^ |
| mN  *0*  *1*  *2* | 20 (57.1%)  5 (14.3%)  10 (28.6%) | 175 (83.3%)  18 (8.57%)  17 (8.10%) | p=0.001^b^ |

Supplementary table 6 – Univariate Cox regression analysis: all patients, pre-treatment factors and disease-free survival

| Characteristic | HR (95% CI) | p  Corrected α=0.005 |
| --- | --- | --- |
| Age (year) | 1.00 (0.99-1.01) | 0.996 |
| Gender  *female*  *male* | *Reference*  1.53 (0.90-2.59) | *Reference*  0.116 |
| Cell subtype  *intestinal*  *diffuse*  *mixed* | *Reference*  1.88 (1.16-3.04)  0.98 (0.47-2.06) | *Reference*  0.010  0.963 |
| Grade of differentiation  *well*  *moderate*  *poor* | *Reference*  0.86 (0.24-3.01)  1.51 (0.47-4.84) | *Reference*  0.809  0.487 |
| Site  *proximal*  *body*  *distal*  *linitis* | *Reference*  1.07 (0.54-2.13)  0.95 (0.53-1.72)  1.17 (0.27-5.12) | *Reference*  0.846  0.870  0.836 |
| T stage  *x/1*  *2/3*  *4* | *Reference*  1.96 (1.02-3.76)  1.91 (0.81-4.50) | *Reference*  0.043  0.140 |
| N stage  *0*  *≥1* | *Reference*  1.10 (0.67-1.79) | *Reference*  0.710 |
| SUVmax (median; IQR) | 0.99 (0.96-1.02) | 0.519 |
| mN  *0*  *1*  *2* | *Reference*  2.10 (1.07-4.10)  3.58 (1.41-9.08) | *Reference*  0.031  0.007 |
| NAC  *No*  *Yes* | *Reference*  2.31 (1.42-3.75) | *Reference*  <0.001 |

NAC=neoadjuvant chemotherapy

Supplementary table 7 – Univariate Cox regression analysis: post-treatment factors and disease-free survival

| Characteristic | HR (95% CI) | p  α=0.006 |
| --- | --- | --- |
| pTR (if NAC)  *Mandard 4/5*  *Mandard 1-3* | *Reference*  0.23 (0.110.47) | *Reference*  <0.001 |
| Grade  *Well*  *Moderate*  *Poor* | *Reference*  1.51 (0.21-11.0)  2.09 (0.51-8.57) | *Reference*  0.773  0.307 |
| pT (TNM7)  *0/1*  *2/3*  *4* | *Reference*  3.74 (1.67-8.35)  8.23 (3.55-19.0) | *Reference*  0.001  <0.001 |
| pN (TNM7)  0  1  2  3 | *Reference*  2.91 (1.50-5.62)  2.83 (1.35-5.91)  4.99 (2.78-8.96) | *Reference*  0.002  0.006  <0.001 |
| pV  *0*  *1* | *Reference*  2.60 (1.64-4.12) | *Reference*  <0.001 |
| pL  *0*  *1* | *Reference*  2.04 (1.26-3.30) | *Reference*  0.004 |
| Resection margin  *0*  *1* | *Reference*  1.51 (0.61-3.75) | *Reference*  0.377 |
| pN mN  *pN0 mN0*  *pN1 mN0*  *pN1 mN1* | *Reference*  2.90 (1.46-5.74)  3.94 (1.38-11.2) | *Reference*  0.002  0.010 |

NAC=neoadjuvant chemotherapy

Supplementary table 8 – Multivariate Cox regression analysis: pre- and post-treatment factors and disease-free survival in patients receiving chemotherapy

| Characteristic | HR (95% CI) | p |
| --- | --- | --- |
| Lauren classification  *Intestinal*  *Diffuse*  *Mixed* | *Reference*  3.53 (1.71-7.29)  1.00 (0.36-2.75) | *Reference*  <0.001  1.000 |
| pTR (if NAC)  *Mandard 4/5*  *Mandard 1-3* | *Reference*  0.27 (0.11-0.63) | *Reference*  0.003 |
| pT (TNM7)  *0/1*  *2/3*  *4* | *Reference*  0.26 (0.03-2.17)  1.48 (0.72-3.04) | *Reference*  0.213  0.290 |
| pN (TNM7)  0  1  2  3 | *Reference*  1.88 (0.70-5.00)  1.99 (0.72-5.21)  1.94 (0.86-4.58) | *Reference*  0.209  0.187  0.108 |
| pV  *0*  *1* | *Reference*  1.40 (0.55-3.57) | *Reference*  0.479 |
| pL  *0*  *1* | *Reference*  0.35 (0.13-0.93) | *Reference*  0.035 |
| mN  *mN0*  *mN1* | *Reference*  2.25 (0.97-5.23) | *Reference*  0.058 |

Supplementary table 9 – Multivariate Cox regression analysis: pre- and post-treatment factors and disease-free survival in patients not receiving chemotherapy

| Characteristic | HR (95% CI) |  |
| --- | --- | --- |
| Age | 1.07 (1.01-1.14) | 0.028 |
| Gender  *Female*  *Male* | *Reference*  3.55 (0.76-16.7) | *Reference*  0.109 |
| pT (TNM7)  *0/1*  *2/3*  *4* | *Reference*  3.13 (0.80-12.2)  0.700 (0.11-4.36) | *Reference*  0.101  0.702 |
| pN (TNM7)  0  1  2  3 | *Reference*  4.28 (1.10-16.7)  4.53 (0.94-21.9)  8.22 (1.68-40.2) | *Reference*  0.036  0.061  0.009 |
| pV  *0*  *1* | *Reference*  0.64 (0.19-2.59) | *Reference*  0.535 |
| pL  *0*  *1* | *Reference*  0.85 (0.24-2.98) | *Reference*  0.803 |
| mN  *0*  *1* | *Reference*  4.90 (0.98-24.5) | *Reference*  0.053 |
| pN mN  *pN0 mN0*  *pN1 mN0*  *pN1 mN1* | *Reference*  2.90 (1.46-5.74)  3.94 (1.38-11.2) | *Reference*  0.002  0.010 |
